# Overall survival following treatment of central nervous system meningeal melanocytomas: Insights from the national cancer database (NCDB)

**DOI:** 10.1016/j.bas.2025.105922

**Published:** 2025-12-29

**Authors:** Victor Gabriel El-Hajj, Jad El Choueiri, Flavio Vasella, Victor E. Staartjes, Mohamad Bydon, Adrian Elmi-Terander

**Affiliations:** a Department of Clinical Neuroscience, Karolinska Institutet, Stockholm, Sweden; b Humanitas University, School of Medicine, Pieve Emanuele, Milan, Italy; c Machine Intelligence in Clinical Neuroscience & Microsurgical Neuroanatomy (MICN) Laboratory, Department of Neurosurgery, Clinical Neuroscience Center, University Hospital Zurich, University of Zurich, Frauenklinikstrasse 10, CH-8091, Zurich, Switzerland; d Department of Neurosurgery, Mayo Clinic, Rochester, MN, USA; e Capio Spine Center Stockholm, Löwenströmska Hospital, Upplands-Väsby, Sweden

**Keywords:** Melanocytoma, Melanocytic tumor, Central nervous system, Brain, Spine, Survival

## Abstract

**Introduction:**

Central nervous system (CNS) melanocytomas are rare, pigmented tumors derived from leptomeningeal melanocytes. Although generally benign, they can exhibit locally aggressive behavior and recur. Despite increasing recognition, data on their clinical outcomes and optimal management remain limited.

**Research question:**

This study aimed to evaluate the survival outcomes of patients with CNS melanocytomas, using a large national registry, and to explore the prognostic relevance of tumor location and treatment modalities.

**Methods:**

We queried the National Cancer Database (NCDB) for cases of CNS melanocytomas diagnosed between 2004 and 2017. Patient demographics, tumor characteristics, treatment details, and survival outcomes were collected. Kaplan-Meier survival analysis was used to study overall survival (OS).

**Results:**

A total of 143 patients with CNS melanocytomas were identified, including 58 spinal (40.6 %), 49 intracranial (34.3 %), 36 tumors of unspecified location (25.2 %). The median age at diagnosis was 59 years, with males comprising 48.3 % of the cohort. Gross total resection (GTR) was reported in 28 patients (19.6 %), while adjuvant radiotherapy was performed in 51 patients (35.7 %). The 1- and 5-year OS rates were approximately 80 % and 50 %, respectively. There were no significant differences in OS based on sex, age, tumor location, extent of resection, or use of adjuvant radiotherapy (p ≥ 0.05).

**Discussion and conclusion:**

Despite advances in surgical techniques and radiation therapy, the optimal management of CNS melanocytomas remains an area of ongoing investigation. Since our findings failed to demonstrate a survival benefit from GTR or the use of adjuvant radiotherapy, future prospective studies should focus on refining treatment indications.

## Highlights

• CNS melanocytomas show a 1- and 5-year OS of ∼80 % and ∼50 % respectively, with no differences based on tumor location, age, or sex.
• Neither extent of resection nor adjuvant radiotherapy significantly impacted overall survival.
• Findings underscore the rarity and heterogeneity of CNS melanocytomas, emphasizing the role of prospective and molecular-based studies.

## 1 Introduction

Melanocytomas were first described in the early 20th century, and the term “melanocytoma” was introduced to differentiate these benign tumors from malignant melanomas. Historically, these tumors were often misdiagnosed as malignant melanomas due to the presence of pigment cells and their pigmented appearance. Early literature, such as the study by Brat et al., in 1999 studied the features of melanocytomas, highlighting their benign nature and favorable prognosis following surgical resection (Brat et al., 1999). Clarke et al. (1998) reviewed historical cases and emphasized the importance of distinguishing melanocytomas from other pigmented central nervous system (CNS) tumors based on histological and clinical features (Clarke et al., 1998).

Melanocytomas are benign tumors originating from melanocytes in the leptomeninges. They represent part of a spectrum of primary melanocytic neoplasms of the CNS, which also includes malignant melanoma and intermediate-grade melanocytic tumors (Brat et al., 1999). Melanocytomas are characterized as well-differentiated, solitary leptomeningeal tumors. Unlike malignant melanomas, which are marked by increased mitotic activity, pleomorphism, necrosis, and frequent invasion of the CNS, melanocytomas typically exhibit a more benign behavior. This differentiation can also be aided by molecular analysis, as GNAQ and GNA11 mutations are common in melanocytomas but not in metastatic melanomas and diffuse tumors, which instead tyically harbor NRAS and BRAF mutation (Wang et al., 2013).

These tumors have been reported to have an incidence of 1 in 10 million people a year, accounting for 0.06 %–0.1 % of brain tumors (Clampitt et al., 2024). They can occur anywhere in the CNS, but most frequently in the posterior fossa and spinal canal (Lee et al., 2017). On magnetic resonance imaging, melanocytomas typically appear iso-to hyperintense on T1-weighted images, hypointense on T2-weighted images, and homogenously enhancing on contrast imaging, though this can vary (Grosshans et al., 2020).

Nevertheless, the literature generally agrees that magnetic resonance imaging (MRI) alone is insufficient for diagnosing melanocytomas, as their appearance can vary depending on the degree of tumor melanization (Yang et al., 2016; Hamasaki et al., 2002; Muthappan et al., 2012). A definitive diagnosis is made through histopathology, which typically reveals melanin-containing, S100-positive cells, along with a low proliferation index and minimal mitotic activity (Grosshans et al., 2020).

Despite their generally benign nature, CNS melanocytomas can recur. In a study by Prasad et al. found that the recurrence rates were 20 % for GTR and 42 % for subtotal resection in a review analyzing 109 cases (Prasad and Divya, 2022). The recurrence rate is reported to be influenced by extent of resection, anatomical location and other factors. Therefore, long-term follow-up is essential to monitor for potential recurrence and malignant transformation (Prasad and Divya, 2022).

Despite some knowledge on melanocytomas, the tumor's response to treatment and the factors influencing survival remain underexplored. This study hence aimed to review all cases of CNS melanocytomas in a large nationwide US-based database, with a focus on the impact of tumor location and treatment approach on overall survival.

## 2 Methods

The study was performed in accordance with all ethical guidelines. In addition, this study was conducted using de-identified data from a publicly available, purchase-accessible database, and therefore did not require institutional review board approval or individual patient consent due to its retrospective design and the absence of identifiable personal information. The need for ethical approval was hence waived by the IRB. The National Cancer Database (NCDB) which captures close to 70 % of new cancer diagnoses in the United States was queried for patients with primary melanocytomas between 2004 and 2017 (Mallin et al., 2019; Battistin et al., 2024; El-Hajj et al., 2025a, 2025b, 2025c). Using the International Classification of Diseases for Oncology, 3rd Edition (ICD-O-3), the codes 8726 and 8725 are applied, along with the appropriate identifiers for the CNS as the primary site. The STROBE guidelines were used and followed during the conduction of this study.

### 2.1 Variables and primary outcome

The NCDB dataset was used to collect data on patient baseline characteristics, tumor details, treatment information, and survival outcomes. Baseline characteristics included sex, age, ethnicity, race, insurance status, income quartile, distance to the treatment facility, and the Charlson-Deyo comorbidity index. Tumor-related data such as histological subtype, metastatic status, and tumor size (in mm) at diagnosis were also recorded. Specific treatment details comprised the extent of tumor resection, type of radiotherapy, number of fractions, radiation doses (cGy), as well as the time to start and duration of radiotherapy. Overall survival was the primary outcome of interest in this study. Patients with incomplete outcome data were excluded from the analysis.

### 2.2 Statistics

The normality of continuous datapoints was evaluated using the Shapiro-Wilk test. As most continuous data significantly deviated from a normal distribution pattern, median and interquartile range (IQR) were presented. Categorical data as frequencies (n) and percentages (%). Kaplan-Meier survival analyses were conducted to compare overall survival rates between groups. Statistical significance of the survival analysis was assessed using the Log-Rank test. All analyses were conducted on R statistical software, version 4.4.3. The threshold for statistical significance was set at p < 0.05.

## 3 Results

### 3.1 Baseline patient characteristics

**Fig. 1:**
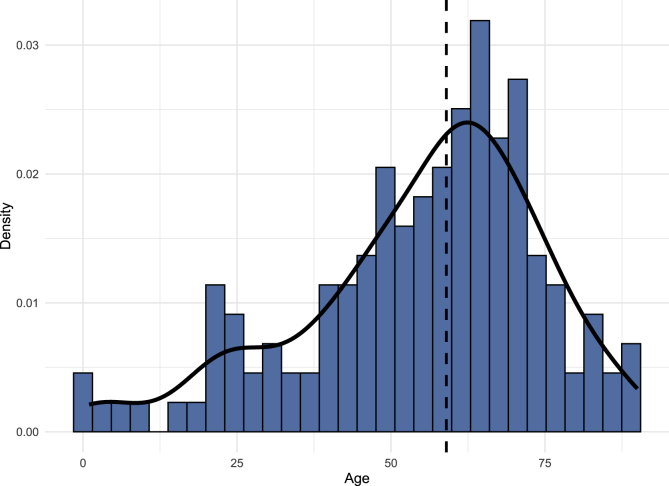
Density histogram illustrating the incidence of melanocytoma across age.

A total of 143 patients with melanocytomas were included in the study, with 58 spinal and 49 intracranial tumors (Table 1). Thirty-six of the cases involved CNS melanocytomas of unknown origin (either intracranial or spinal). The peak incidence of melanocytoma occurred during the 7th decade of life (Fig. 1). The median age at diagnosis for the overall cohort was 59 years. Patients with spinal melanocytomas had a higher median age of 61 years compared to 56 years for patients with intracranial ones (**Supplementary Table A**). Male sex constituted 48 % of the total cohort, with comparable proportions in spinal and intracranial groups. The absolute majority of patients were White (95 %) and non-Hispanic (95 %), with no differences based on tumor location.

**Table 1:** Baseline characteristics of patients with melanocytoma that are included in the NCDB.

Characteristic	Overall, N = 143
**Age**	59.0 (45.5, 68.5)
**Male sex**	69 (48 %)
**Race**	
White	135 (95 %)
Black	5 (3.5 %)
Other	3 (2 %)
**Hispanic ethnicity**	7 (4.9 %)
**Charlson-Deyo comorbidity index**	
0	103 (72 %)
1	21 (15 %)
2	11 (7.7 %)
3	8 (5.6 %)
**Largest tumor diameter (mm)**	30.0 (20.0, 40.0)
**Surgical treatment**	
Surgery, Unspecified	68 (48 %)
Surgery, Gross Total Resection	18 (13 %)
Surgery, Subtotal Resection	18 (13 %)
Biopsy only	39 (27 %)
**Radiation therapy**	52 (36 %)
**Time from diagnosis to radiotherapy (days)**	63.0 (41.0, 147.0)
**Radiotherapy modality**	
External beam radiation	24 (46 %)
Stereotactic radiosurgery	15 (29 %)
Intensity modulated therapy	7 (13 %)
Conformal radiation therapy	6 (12 %)
**Total duration of radiotherapy (days)**	35.0 (4.0, 40.0)
**Total radiation dose (in cGy)**	14.0 (8.8, 17.3)
**Chemotherapy**	9 (6.3 %)
**Total length of hospital stays (days)**	5.0 (4.0, 8.0)
**30-day unplanned readmission**	5 (3.5 %)
**30-day mortality**	5 (3.5 %)
**90-day mortality**	9 (6.3 %)

Socioeconomic factors, such as insurance status, income, and residential area, were also similar between the groups. Private insurance was the most common coverage (54 %), followed by Medicare (34 %), Medicaid (7.0 %). Only 2.8 % of patients were reportedly uninsured. Both cohorts demonstrated a fairly even distribution across income quartiles. With regards to residential area, most patients resided in metropolitan regions (83 %).

Comorbidity scores, as assessed by the Charlson-Deyo comorbidity index, showed that the majority of patients (72 %) had no comorbidities, with no notable difference between spinal and intracranial groups. Higher comorbidity scores were less frequent across both cohorts.

The median largest tumor diameter was 30 mm, with spinal tumors being slightly smaller (24 mm) than intracranial tumors (33 mm) (Table 1).

### 3.2 Treatment

Most patients received surgical treatment however the extent of resection was unspecified in (48 %). Gross total resection was performed for 20 % of spinal tumors and 17 % of intracranial tumors. Subtotal resection was performed for 18 % of spinal and 8 % of intracranial tumors (8 %). Biopsy alone was performed in 42 % of intracranial tumors and 35 % of spinal tumors. The median time from diagnosis to surgery was similar between groups, with an overall median of 0.5 days.

Radiation therapy was administered in 36 % of cases, with similar proportions in spinal and intracranial groups. However, the modality of radiation therapy differed. External beam radiation therapy was more commonly used for spinal melanocytomas (54 %) than intracranial cases (30 %), while stereotactic radiosurgery was more frequently employed for intracranial tumors (52 %) than spinal tumors (12 %). The median total radiation dose was 14 cGy, with no significant difference between spinal and intracranial tumors. Chemotherapy use was limited, with only 6 % of the overall cohort receiving it (Table 1).

### 3.3 Outcomes

Hospital stays were consistent across groups, with a median length of 5 days in both spinal and intracranial cases. Thirty-day unplanned readmission rates were low (3.5 %), with no difference between groups. Mortality rates were similarly low, with 30-day mortality at 3.5 % and 90-day mortality at 6 %, showing no notable variation between spinal and intracranial tumors (Table 1).

### 3.4 Overall survival

**Fig. 2:**
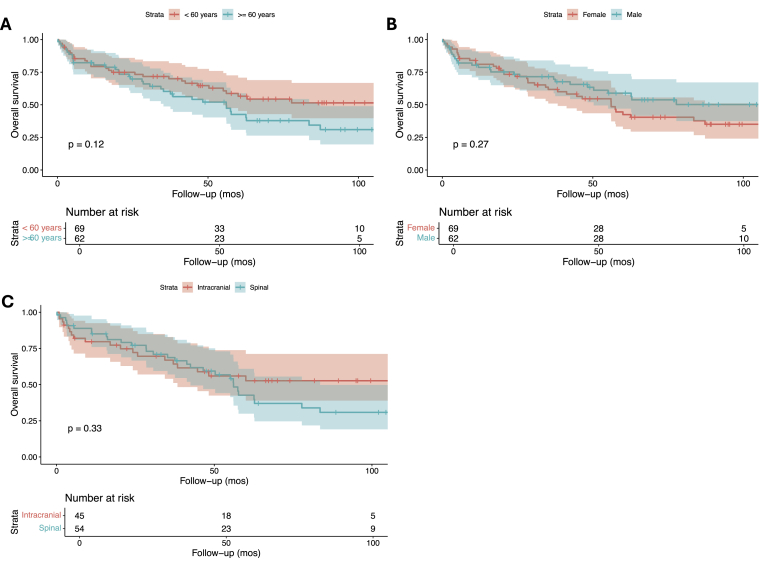
Overall survival of patients with melanocytomas based on age (A), sex (B), and tumor location (C).

**Fig. 3:**
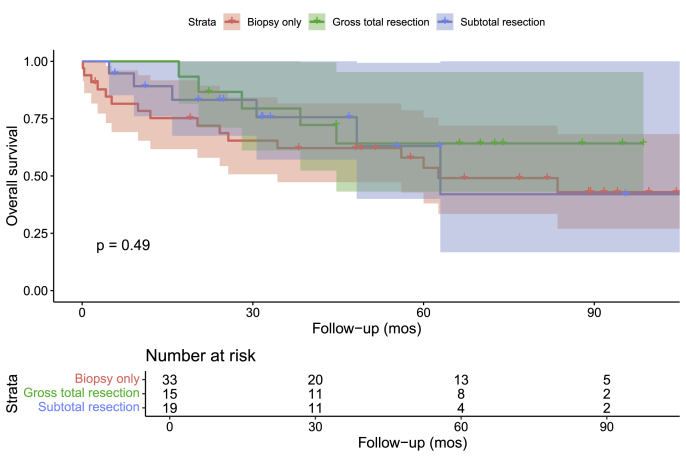
Overall survival of patients with melanocytomas based on the extent of tumor resection.

**Fig. 4:**
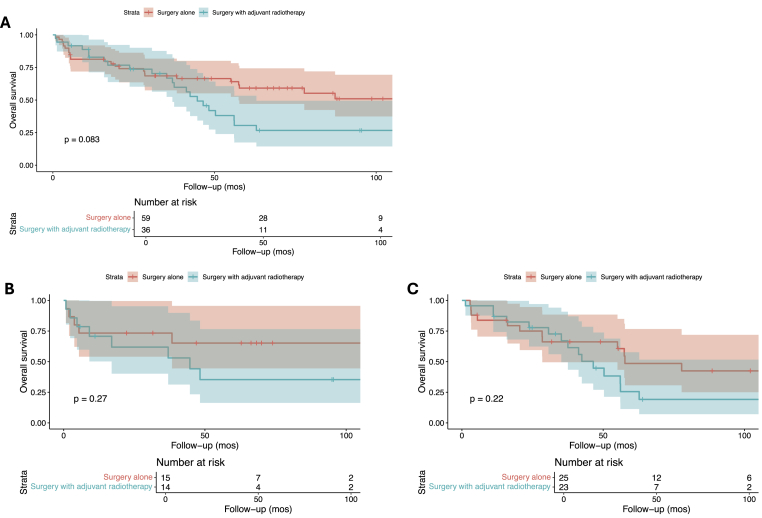
Overall survival of patients with melanocytomas following surgery with versus without adjuvant radiotherapy in the overall cohort (A), among patients with intracranial (B), and spinal melanocytomas (C).

Kaplan Meier survival analysis revealed a 1-year overall survival rate of around 80 % and a 5-year overall survival rate of around 50 % in patients treated for CNS melanocytomas, including both intracranial and spinal ones (**Supplementary Figure A**). There were no differences in overall survival based on the age of onset (<60 vs. ≥60 years), sex, or primary tumor site (Fig. 2A, B, and 2C; p ≥ 0.05). There were no significant overall survival differences in terms of the extent of tumor resection (Fig. 3; p = 0.49). Similarly, compared to surgery alone, adjuvant radiotherapy with surgery could not be associated with any significant overall survival benefit in the total cohort (Fig. 4A; p = 0.083), among patients with intracranial (Fig. 4B; p = 0.27), or spinal melanocytomas (Fig. 4C; p = 0.22).

## 4 Discussion

CNS melanocytomas constitute a spectrum of rare tumors. Their rarity is reflected through the relatively limited number of studies discussing their response to treatments.

Our study examines the overall survival (OS) following treatment of brain and spinal melanocytomas in 143 patients from the National Cancer Database (NCDB), aiming to provide insights into understanding the prognostic implications of distinct anatomical locations and treatment approaches on survival outcomes.

We analyzed 58 spinal, 49 intracranial, and 36 melanocytomas of unknown origin. The median age at diagnosis for the cohort was 59 years, with a slightly older median age of 61 years for the spinal group and 56 years for the intracranial group. These numbers are notably higher than reported in previous studies. For instance, in a review of 201 published cases by Ricchizzi et al. (2022), the authors found a median age of 38, which may be the result of publication bias. In another work by Rades et al. (2004). On 89 patients, the median age of the included patients was 45. The higher median age observed in our study may also be influenced by selection bias inherent to the NCDB database.

Males and females were equally represented across the cohort, with a ratio comparable to the one shown in other large studies (Grosshans et al., 2020). Spinal tumors were smaller in size as compared to intracranial ones (24 mm vs. 33 mm). This may be due to earlier symptom onset in the confined spinal canal, leading to their earlier detection at smaller sizes.

The current literature seems to agree that prognosis of these tumors is variable (Küsters-Vandevelde et al., 2015). Our survival analysis showed an overall survival of 80 % around 1 year, and 50 % around 5 years in patients treated for CNS melanocytomas. Given the absence of tumor WHO grade data in the NCDB registry, it is reasonable to infer that these mortality rates likely reflect a mixed population of both benign and intermediate-grade CNS melanocytomas, with the latter likely contributing to lower overall survival rates (Zembowicz et al., 2004; Perrini et al., 2010; Ferracini et al., 1980).

Nonetheless, these numbers aligned with those presented by Tep et al. (Sahgal et al., 2021) who showed a 5-year survival of 48 %. Interestingly, the group emphasized that income disparities significantly affected survival outcomes.

Similarly, in a review by Antkowiak et al. (Koelsche et al., 2015), the 1, 2, 3 and 4-year overall survival rates were 80 %, 71 %, 71 % and 50 % respectively, in pediatric patients diagnosed with CNS melanocytomas.

In the current study, no notable difference between intracranial and spinal tumors could be found, potentially indicating similar behavior of the tumor counterparts.

According to previous literature, an important determinant of outcomes in patients with melanocytomas is the degree of surgical resection (Hamasaki et al., 2002; Rades et al., 2004). Numerous studies with limited level of evidence suggest that gross total resection (GTR) is the preferred treatment, while subtotal resection (STR) yields better outcomes when combined with radiation therapy (RT) (Ricchizzi et al., 2022; Pellerino et al., 2024; Rades et al., 2024).

For instance, a 2024 review by Rades et al. (2024) evaluated the role of extent of tumor resection and adjuvant radiotherapy for meningeal melanocytomas in terms of both overall survival and local control. The authors found that GTR was significantly superior to STR and that RT significantly improved outcomes following STR. These findings have also been suggested in earlier reviews (Rades et al., 2004).

It is, however, important to note that these reviews primarily analyzed previously published literature, mainly consisting of case reports and small series, which introduces significant bias. Interestingly, the current study could not associate extent of tumor resection with overall survival. Nonetheless, while surgery was performed in most of the included patients, the extent of resection was only specified in about half of the cases, limiting the power of analysis.

Regardless, GTR was achieved in 20 % of spinal tumors and 17 % of intracranial tumors, highlighting that despite its potential benefits for progression-free and overall survival, it is only achieved in a minority of patients.

Consequently, adjuvant radiotherapy has been employed as a complementary approach to enhance disease control rates, particularly in patients for whom GTR may not be feasible. In the current cohort, RT was administered at similar rates in both spinal and intracranial groups, with external beam radiation therapy (EBRT) was more commonly used for spinal melanocytomas (54 % vs. 30 %), and stereotactic radiotherapy (SRT) in intracranial cases (52 % vs. 12 %).

EBRT involves directing radiation typically over multiple sessions and is usually suitable for larger or more diffuse tumors. The goal is to achieve local control while minimizing the risk of radiation myelopathy. Studies have shown that doses of 50–52 Gy in 1.8 Gy fractions are effective in improving local control and overall survival after incomplete resection (ITR) of spinal melanocytomas (Rades and Schild, 2006). In contrast, SRT delivers precise, high-dose radiation in fewer fractions, targeting well-defined tumor regions with greater accuracy (Sahgal et al., 2021). Hamasaki et al. for example, highlighted improved outcomes following subtotal resection in conjunction with gamma-knife radiosurgery in meningeal melanocytoma (Hamasaki et al., 2002). SRT has also shown to be effective in managing recurrent or residual intracranial melanocytomas, with studies showing local tumor control rates of 76.9 % (Dar et al., 2022).

In their review, Rades et al. (2024) found that STR with adjuvant RT only provided local control, but no overall survival benefits when compared to STR alone, suggesting a progression-free survival benefit associated with this modality. Similarly, in our study radiation therapy could not be associated with any overall survival benefits. However, since the NCDB database does not account for any other endpoints, the impact of the use of adjuvant radiotherapy on progression-free survival and local tumor control could not be verified. Solid evidence in the form of prospective interventional studies is needed to better answer this question. This is primarily because patients with more severe disease and more aggressive tumors are more likely to receive radiotherapy, potentially introducing a bias associated with retrospective studies. This is reflected in the current study, with the observed trend of slightly increased mortality among those undergoing adjuvant radiotherapy.

Generally, favorable short-term postoperative outcomes were observed for both intracranial and spinal melanocytomas, as the average hospital stay was 5 days, with low unplanned readmission and mortality rates.

Beyond clinical and pathological factors, emerging molecular and genetic insights are providing a deeper understanding of these tumors and their biological behavior. While these aspects were lacking from the current analysis, studies have recently shown that these parameters resonate well with the prognosis following management of CNS melanocytomas and larger prospective studies should however further explore the characteristics of these tumors.

For instance, commonly found mutations associated with CNS melanocytomas include GNAQ and GNA11, found in circumscribed melanocytic tumors. These mutations were however less common in diffuse primary meningeal melanocytic tumors, which harbor NRAS and BRAF mutations (Küsters-Vandevelde et al., 2015; Koelsche et al., 2015). The absence of oncogenic mutations is associated with their benign nature, and these characteristics become important to note as intermediate-grade melanocytomas, which exhibit features between benign melanocytomas and malignant melanomas, can have a more aggressive course, including higher recurrence rates and a higher potential of malignant transformation (Koelsche et al., 2015). Additional mutations in SF3B1, EIF1AX, and BAP1 have been identified in several cases, and can help distinguish these tumors from other melanocytic lesions (Küsters-Vandevelde et al., 2015; Pellerino et al., 2024; Ghaith et al., 2024).

### 4.1 Limitations

Our analysis remains limited by several factors. Firstly, the retrospective nature of our study and of the NCDB dataset might have introduced selection bias and limited the ability to control confounding factors. The NCDB relies only on Commission of Cancer-approved hospitals (Tep et al., 2021; Bilimoria et al., 2009), and also lacks information on factors that affect progression-free survival (Boffa et al., 2017). Detailed information on tumor WHO grade in most patients as well as extent of tumor resection in almost half of the patients was absent from the database. The high proportion of cases with unspecified resection extent could have obscured the actual impact of GTR compared to STR. Likewise, the absence of WHO grade data may have introduced unmeasured confounding in the survival analysis. In conclusion, interpretation of survival outcomes should be made with caution, as nearly half of the cohort had an unspecified extent of resection, which may have limited the ability to detect true associations between surgical extent, adjuvant radiotherapy, and overall survival. Additionally, the lack of available WHO grading and molecular characterization precluded granular and adequately powered differentiation between grades, which may have masked true differences in clinical behavior and survival outcomes.

## 5 Conclusion

Findings from the current study indicate a 1-year overall survival rate of around 80 % and a 5-year overall survival rate of around 50 % in patients treated for CNS melanocytomas. Overall survival was not affected by age, sex, primary tumor location, extent of tumor resection, or use of adjuvant radiation therapy. Despite advances in surgical techniques and radiation therapy, the optimal management of CNS melanocytomas remains an area of ongoing investigation. Since our findings did not demonstrate a survival benefit from adjuvant radiotherapy, future prospective studies should focus on refining indications for adjuvant treatment. Additionally, the advancements of molecular classification and characterization of these tumors could guide further therapies. Finally, these conclusions should be interpreted with caution given the limitations related to missing data and the registry-based nature of the study.

## Declaration of competing interest

The authors declare that they have no known competing financial interests or personal relationships that could have appeared to influence the work reported in this paper.

## Appendix A Supplementary data

Supplementary **Figure A. Overall survival of patients with melanocytomas.**Multimedia component 1
