# Supplementary figures and images for: Overall survival following treatment of central nervous system meningeal melanocytomas: Insights from the national cancer database (NCDB)
Source: Brain Spine. 2025 Dec 29;6:105922. doi: 10.1016/j.bas.2025.105922 (PMC12804625; doi:10.1016/j.bas.2025.105922)

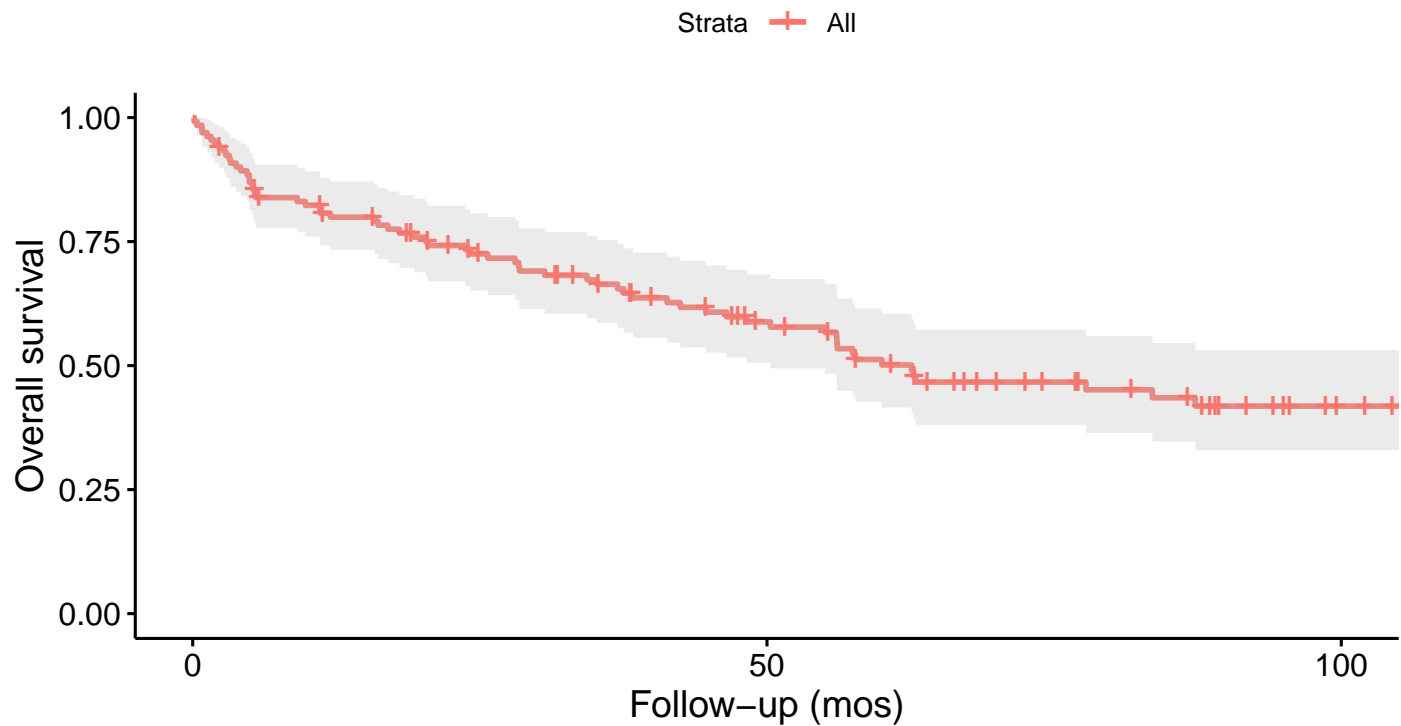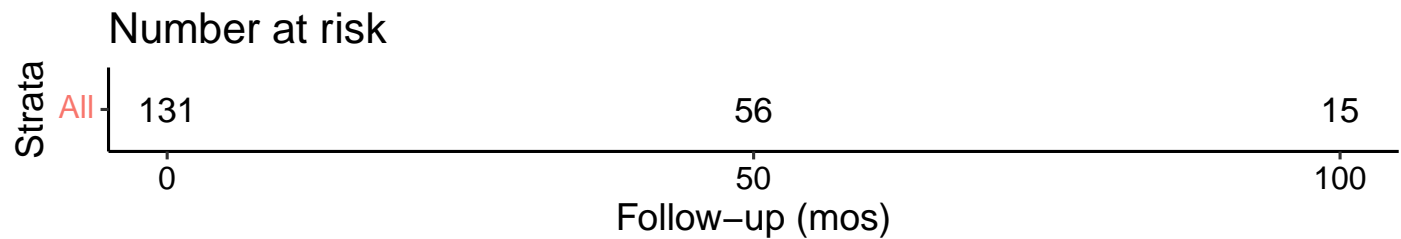

Supplement: Supplementary file 1 — Supplementary Figure A. Overall survival of patients with melanocytomas.Multimedia component 1 [file mmc1.pdf]
