# Supplementary material for: Overall survival following treatment of central nervous system meningeal melanocytomas: Insights from the national cancer database (NCDB)
Source: Brain Spine. 2025 Dec 29;6:105922. doi: 10.1016/j.bas.2025.105922 (PMC12804625; doi:10.1016/j.bas.2025.105922)
Supplement: Multimedia component 2 [file mmc2.docx]

**Table 1.** Baseline characterisitics of patients with melanocytoma that are included in the NCDB.

| **Characteristic** | **Spinal cord**,  N = 58 | **Brain**,  N = 49 |
| --- | --- | --- |
| **Age** | 60.5 (51.3, 69.8) | 56.0 (39.0, 70.0) |
| **Male sex** | 31 (53%) | 24 (49%) |
| **Race** |  |  |
| White | 58 (100%) | 45 (92%) |
| Black | 0 (0%) | 3 (6.1%) |
| Other | 0 (0%) | 1 (2.0%) |
| **Hispanic ethnicity** | 0 (0%) | 5 (10%) |
| **Insurance status** |  |  |
| Private insurance | 31 (53%) | 26 (53%) |
| Medicare | 21 (36%) | 19 (39%) |
| Medicaid | 4 (6.9%) | 4 (8.2%) |
| Uninsured | 1 (1.7%) | 0 (0%) |
| Other | 1 (1.7%) | 0 (0%) |
| **Income quartiles** |  |  |
| 1 | 9 (16%) | 10 (20%) |
| 2 | 14 (24%) | 9 (18%) |
| 3 | 13 (22%) | 16 (33%) |
| 4 | 22 (38%) | 14 (29%) |
| **Residential area** |  |  |
| Metro | 46 (79%) | 40 (82%) |
| Urban | 11 (19%) | 8 (16%) |
| Rural | 1 (1.7%) | 1 (2.0%) |
| **Charlson-Deyo comorbidity index** |  |  |
| 0 | 42 (72%) | 41 (84%) |
| 1 | 7 (12%) | 4 (8.2%) |
| 2 | 6 (10%) | 2 (4.1%) |
| 3 | 3 (5.2%) | 2 (4.1%) |
| **Largest tumor diameter (mm)** | 24.5 (20.0, 38.8) | 33.0 (22.0, 38.0) |
| **Surgical treatment** |  |  |
| Surgery, Unspecified | 13 (27%) | 12 (33%) |
| Surgery, Gross Total Resection | 10 (20%) | 6 (17%) |
| Surgery, Subtotal Resection | 9 (18%) | 3 (8.3%) |
| Biopsy only | 17 (35%) | 15 (42%) |
| **Time from diagnosis to surgery (days)** | 0.0 (0.0, 15.8) | 4.0 (0.0, 18.0) |
| **Radiation therapy** | 27 (47%) | 23 (47%) |
| **Time from diagnosis to radiotherapy (days)** | 58.5 (46.0, 153.8) | 63.0 (37.0, 130.0) |
| **Radiotherapy modality** |  |  |
| External beam radiation | 14 (54%) | 7 (30%) |
| Stereotactic radiosurgery | 3 (12%) | 12 (52%) |
| Intensity modulated therapy | 4 (15%) | 3 (13%) |
| Conformal radiation therapy | 5 (19%) | 1 (4.3%) |
| **Total duration of radiotherapy (days)** | 36.0 (35.0, 40.0) | 5.5 (1.0, 34.8) |
| **Total radiation dose (in cGy)** | 13.5 (13.0, 16.0) | 10.0 (4.5, 19.0) |
| **Chemotherapy** | 1 (1.7%) | 4 (8.2%) |
| **Total length of hospital stays (days)** | 5.0 (4.0, 9.0) | 5.0 (4.0, 7.0) |
| **30-day unplanned readmission** | 1 (1.7%) | 2 (4.1%) |
| **30-day mortality** | 1 (1.7%) | 2 (4.1%) |
| **90-day mortality** | 4 (6.9%) | 3 (6.1%) |
